# Regenerative medicine technologies applied to transplant medicine. An update

**DOI:** 10.3389/fbioe.2022.1015628

**Published:** 2022-09-28

**Authors:** Astgik Petrosyan, Filippo Montali, Andrea Peloso, Antonio Citro, Lori N. Byers, Catherine La Pointe, Mara Suleiman, Alice Marchetti, Eoin P. Mcneill, Allison L Speer, Wai Hoe Ng, Xi Ren, Benedetta Bussolati, Laura Perin, Paolo Di Nardo, Vincenzo Cardinale, Jerome Duisit, Alexandra Rose Monetti, John Richard Savino, Amish Asthana, Giuseppe Orlando

**Affiliations:** ^1^ GOFARR Laboratory for Organ Regenerative Research and Cell Therapeutics in Urology, Saban Research Institute, Division of Urology, Children’s Hospital Los Angeles, Los Angeles, CA, United States; ^2^ Department of General Surgery, di Vaio Hospital, Fidenza, Italy; ^3^ Visceral Surgery Division, University Hospitals of Geneva, Geneva, Switzerland; ^4^ San Raffaele Diabetes Research Institute, IRCCS Ospedale San Raffaele, Milan, Italy; ^5^ Wake Forest School of Medicine, Winston Salem, NC, United States; ^6^ Department of Clinical and Experimental Medicine, University of Pisa, Pisa, Italy; ^7^ Department of Pharmaceutical Sciences, Università del Piemonte Orientale, Novara, Italy; ^8^ Department of Pediatric Surgery, The University of Texas Health Science Center at Houston McGovern Medical School, Houston, TX, United States; ^9^ Department of Biomedical Engineering, Carnegie Mellon University, Pittsburgh, PA, United States; ^10^ Department of Molecular Biotechnology and Health Sciences, University of Turin, Turin, Italy; ^11^ Centro Interdipartimentale per la Medicina Rigenerativa (CIMER), Università Degli Studi di Roma Tor Vergata, Rome, Italy; ^12^ Department of Medico-Surgical Sciences and Biotechnologies, Sapienza University of Rome, Rome, Italy; ^13^ Department of Plastic, Reconstructive and Aesthetic Surgery, CHU Rennes, University of Rennes I, Rennes, France

**Keywords:** regenerative medicine, transplant medicine, cell therapeutics, organ regeneration, tissue engineering

## Abstract

Regenerative medicine (RM) is changing how we think and practice transplant medicine. In regenerative medicine, the aim is to develop and employ methods to regenerate, restore or replace damaged/diseased tissues or organs. Regenerative medicine investigates using tools such as novel technologies or techniques, extracellular vesicles, cell-based therapies, and tissue-engineered constructs to design effective patient-specific treatments. This review illustrates current advancements in regenerative medicine that may pertain to transplant medicine. We highlight progress made and various tools designed and employed specifically for each tissue or organ, such as the kidney, heart, liver, lung, vasculature, gastrointestinal tract, and pancreas. By combing both fields of transplant and regenerative medicine, we can harbor a successful collaboration that would be beneficial and efficacious for the repair and design of *de novo* engineered whole organs for transplantations.

## Background

In 2011, Transplant International published the first manuscript illustrating how regenerative medicine technologies will impact transplant medicine (Orlando et al., 2011). In the years that followed, the manuscript ranked among the ten most downloaded papers from the website of that journal as a testament to the special interest that regenerative medicine generates in the transplant community. A decade later, regenerative medicine has progressed significantly while hurdles that were unknown at that time have been unveiled. At the same time, the transplant community has started investing significantly in regenerative medicine and has undertaken many initiatives to bridge the two fields. For example, transplant conferences are granting more and more visibility to regenerative medicine topics and are allocating relevant space to regenerative medicine-oriented sessions. In 2016, the International Pancreas and Islet Transplant Association (IPITA) launched in collaboration with the Juvenile Diabetes Research Foundation (JDRF) and the Harvard Stem Cell Institute. This is a one-of-a-kind conference fully dedicated to the application of stem cell technologies to beta-cell replacement; in 2020, this conference series celebrated its third edition despite the COVID pandemic. In January 2021, the American Society of Transplantation (AST) signed a letter of collaboration with the Tissue Engineering and Regenerative Medicine International Society (TERMIS) with the intent to–as explained in the AST website–bringing “*together experts from both fields on the same stage for the first time, in order to share knowledge and ultimately foster progress in organ bioengineering, regeneration and repair which will shape and define the future of both worlds*” (https://www.myast.org/meetings/ast-termis-webinar-joining-forces-shape-our-mutual-future). What has stemmed so far from this collaboration is a new webinar series featuring speakers from both worlds and whose sessions are available on YouTube (the link https://www.youtube.com/watch?v=Qz21se2VSbs&t=212s relates to the very first edition of the webinar series). On the editorial front, transplant journals are publishing more and more regenerative medicine manuscripts. Transplant societies are establishing committees focusing on regenerative medicine-related topics like cell therapy or organ bioengineering. Some examples: In 2014, AST launched the Transplant Regenerative Medicine Community of Practice. The Cell Transplantation Society rebranded its name as the Cell Transplant and Regenerative Medicine Society. At the same time, while the European Society of Organ Transplantation (ESOT) established the European Cell Therapy and Organ Regeneration Section (ECTORS) in 2019.

This manuscript aims at updating the transplant audience on the recent advances in regenerative medicine that may be pertinent to transplant medicine. These advances will be presented separately by organs.

## Kidney

### Stem cells and their bioproducts for kidney transplant

Mesenchymal stromal cells (MSC) have long been of interest to the kidney transplantation world mainly for their immunomodulatory properties (Podesta et al., 2020). However, aside from their ability to modulate the host immune response, these cells also possess remarkable regenerative, reparative, and angiogenic properties. Their potential medical utility continues to be investigated in about one thousand clinical trials (Pittenger et al., 2019). Initially, MSCs were proposed for cellular therapy, but recently superimposable beneficial effects have been reported using MSC-derived extracellular vesicles (EVs) (Bruno et al., 2019; Correa et al., 2021). These vesicles are nano sized vehicles containing a specific active cargo able to reprogram target cells.

MSCs and their derivatives may have a role in kidney transplantation at mitigating ischemia-reperfusion injury deriving from the stress and tissue damage related to the chain of events donor death>>>procurement surgery>>>organ storage>>>implantation. Strong data demonstrating that MSC may enhance adaptive repair in ischemically damaged human kidneys was provided by Brasile et al. (2019) in an *ex vivo* model of DCD renal allograft preservation. In this study, five pairs of discarded DCD kidneys were treated with 10^8^ MSC or placebo, perfused *ex vivo* for 24 h in a proprietary machine perfusion system, and eventually evaluated for DNA synthesis, cytokine/chemokine synthesis, cytoskeletal regeneration, and mitosis. The authors observed that the study group showed increased synthesis of adenosine triphosphate, a reduced inflammatory response, increased synthesis of growth factors, normalization of the cytoskeleton, and mitosis. More recently, a similar study conducted *ex vivo* reported comparable results using 50 million multipotent adult progenitor cells (MAPC) that are inherently similar to MSC (Thompson et al., 2021). In this report, grafts were perfused at 36.5°C for only 7 h and demonstrated improvement in clinically relevant parameters and injury biomarkers. This notwithstanding, two recently published *in vivo* studies have failed in showing beneficial effect of MSC. In a kidney autotransplantation porcine model, the administration of a much lower dose of MSCs (10 million) during *ex vivo* normothermic machine perfusion (Lohmann et al., 2021a) or after cold storage (Lohmann et al., 2021b) was not followed by any beneficial clinical effect within 2 weeks of observation.

**FIGURE 1:**
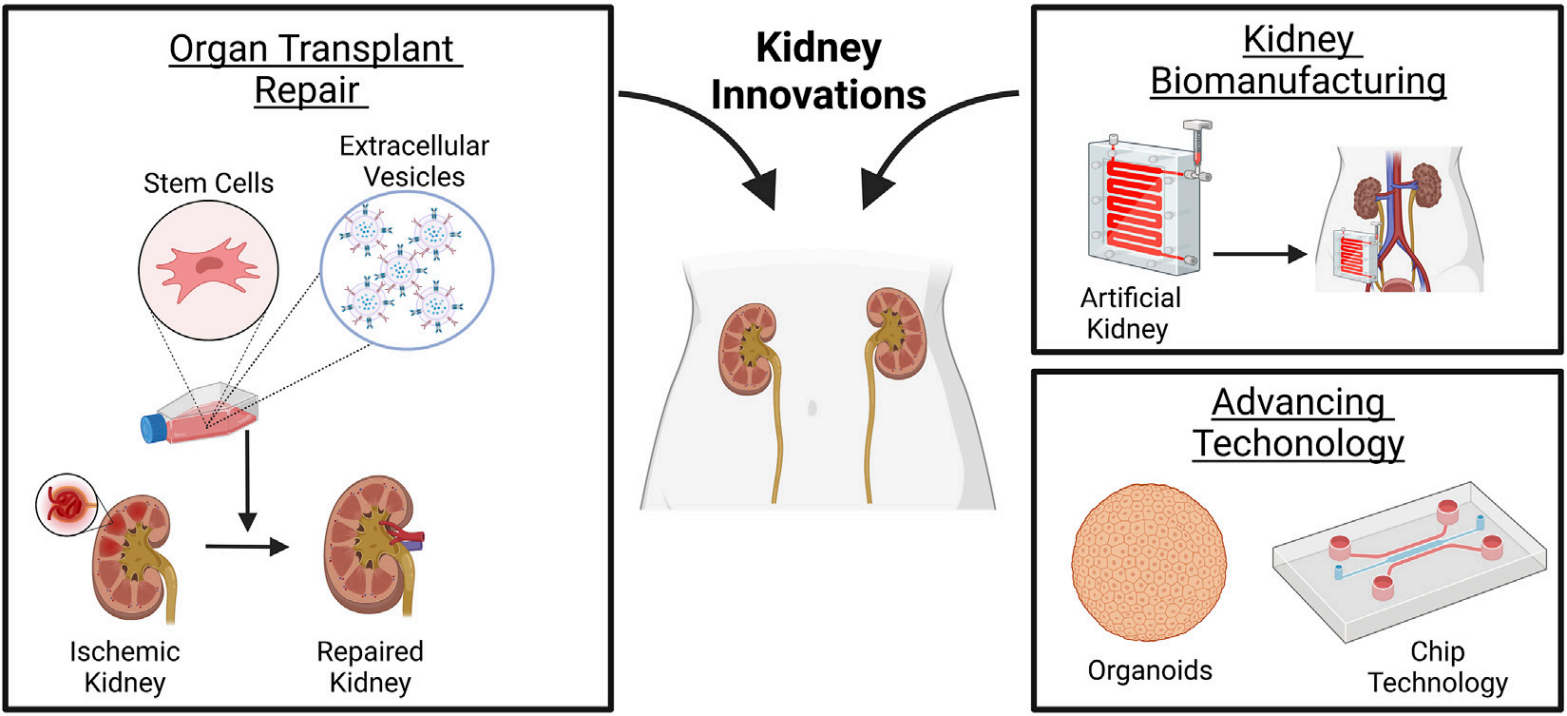
Innovations made in renal medicine. Stem cells or derived EVs may mitigate ischemic renal damage before or after transplantation. The design of artificial kidney devices, although limited in fully mimicking kidney function, i.e., secretion of endocrine and immunologic factors, reabsorption, or metabolism, may allow home dialysis and self-care renal replacement therapy for patients waiting for a transplant. Advancing technology in organoids and chip systems may serve as a platform to study disease mechanisms and perform drug screening studies with high reproducibility for the design of patient-specific therapies. Created with BioRender.com.

Few studies have investigated the possibility of using stem cells or derived EVs to mitigate ischemic renal damage after transplantation (Figure 1). Wu *et al.* demonstrated that human Wharton’s Jelly MSC-EVs mitigated renal damage, ameliorated function, and improved survival when administered intravenously in post-transplant DCD kidneys in rats (Wu et al., 2018). MSC-EVs reduced cell apoptosis and inflammation, as well as promoted cell proliferation. The effects of MSC-EVs were evaluated 2 weeks post-transplantation and demonstrated reduced renal fibrosis and macrophage infiltration upon EV administration, sustaining their beneficial role in the acute and chronic stages (Wu et al., 2018).

Gregorini et al. (2017) proposed using stem cells or their released EVs to supplement the standard perfusion solution. In detail, they performed 20 min of warm ischemia followed by nephrectomy in rats. The explanted kidneys were perfused for 4 h at 4°C in the hypothermic machine perfusion, with the supplement of 3 million MSCs or EVs, released by the same number of cells. Molecular and histological analyses of kidneys immediately post perfusion, treated with MSC or MSC EVs, revealed significantly lower renal damage than control kidneys and showed an up-regulation of energy metabolism enzymes. Moreover, the evaluation of lactate, glucose, and LDH in the effluent fluid indicated extensive use of energy substrate in the presence of MSC and MSC EVs. More recently, the same group showed that EVs delivered during hypothermic oxygenated perfusion into marginal kidneys significantly reduces ischemia-reperfusion injury (Rampino et al., 2022).

### 3D kidney biomanufacturing

Tissue engineering (TE) and advances in three-dimensional bioprinting techniques that use a combination of cells, artificial and natural biomaterial, and biologically active molecules to reconstruct or regenerate damaged tissues or whole organs provide a potential solution to the shortage of transplantable kidneys. However, unlike two-dimensional planar tissue, the complex kidney structure is composed of various cell types in specialized locations on the specific composition of extracellular matrix protein for proper kidney function. Thus, making bioengineering of the kidney for transplantation still challenging. Although challenges still exist in whole organ engineering, the development of wearable hemodialysis devices may serve as a viable novel alternative dialysis technology that can enhance a patient’s freedom and quality of life (Figure 1). Multiple clinical trials have shown wearable artificial kidneys’ benefits and possible pitfalls. Gura et al. (2008) showed in an FDA-approved human trial the design and use of wearable artificial kidneys (WAK). These artificial kidneys were miniaturized, wearable hemodialysis machine, based on dialysate-regenerating sorbent technology. They were designed to be well-tolerated and effective in uremic solute clearance and maintenance of electrolyte and fluid homeostasis for up to 24 h. The University of California, San Francisco, and Vanderbilt University Medical Center have been rigorously developing an implantable artificial kidney (IAK). The group uses a combination of a high-efficiency membrane for hemofiltration with a bioreactor of kidney tubule cells for electrolyte balance (Salani et al., 2018). Improvements are being made to address the pitfalls of artificial kidneys, such as thrombogenicity, excessive carbon dioxide bubbles, device portability, extended service life, reduced replacement of sorbent cartridges, differentiated phenotype maintenance of cultured tubule cells, and cost-effectiveness. Although these devices are limited in fully mimicking kidney function, i.e., secretion of endocrine and immunologic factors, reabsorption, or metabolism, they do allow home dialysis and self-care renal replacement therapy to be more feasible. They may still serve as a viable option for patients waiting for a transplant.

### Organoids and chip technology

In kidney transplant medicine, novel techniques, and technologies such as three-dimensional (3D) cell cultures that incorporate key kidney features can lead to the design of more patient-specific targeted therapies (Figure 1). Kidney organoids, which are 3D cell cultures composed of various cell types, i.e., human pluripotent stem cells (hPSCs) differentiated to kidney cell types, are designed for drug screening, disease modeling, and the generation of tissue for renal replacement. Recently Lawlor et al. (2021) showed that by 3D bioprinting organoids, more manufactured organoids with specific biophysical properties such as size, cell number, and conformation might be generated towards creating uniform patterned kidney tissue sheets. However, limitations still exist in mimicking the filtration barriers and fluid exchange vital for kidney function and responsible for blood filtration with excretion of metabolic waste products and drugs. The use of microfluidic chips, such as glomerulus-on-a-chip (referred to as GOAC) (Petrosyan et al., 2019) and proximal tubule epithelial cells (PTEC) on a chip (Vriend et al., 2018) are shown to recapitulate the functions and structure of the glomerulus. These changes include perm selectivity and active tubular secretions through proximal tubules drug transporters. Current advancements are being made towards addressing the technological limitations regarding the chips’ bi-directionality of the flow and the absence of all kidney cell types needed to mimic the full nephron by generating four-lane chips with a unidirectional flow. Nonetheless, the chip systems serve as a platform to study disease mechanisms and perform drug screening studies with high reproducibility.

## Heart

**FIGURE 2:**
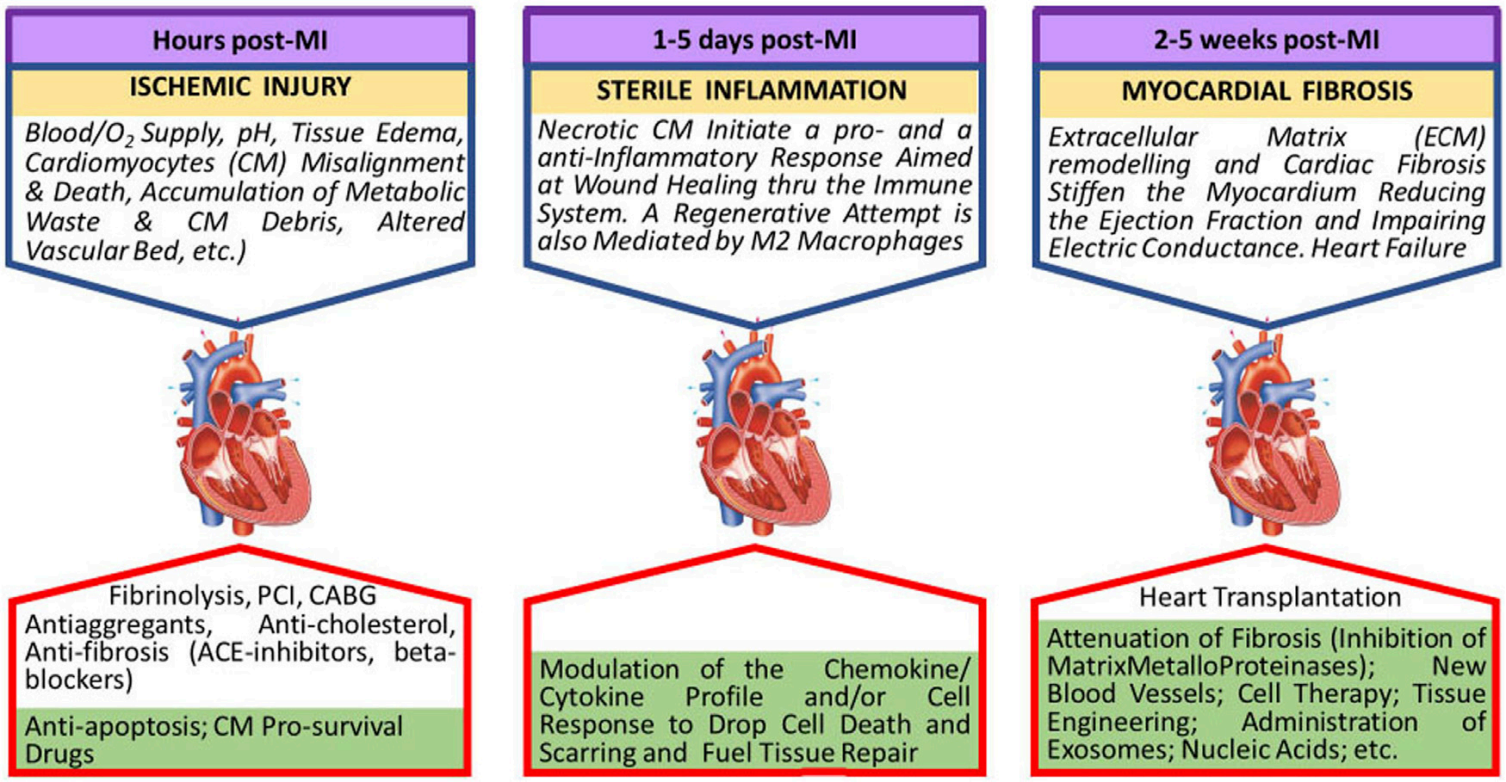
Evolution of the myocardial ischemic damage. In lower green boxes future research directions are indicated.

Cardiac cell therapy is considered the only cure for cardiovascular disease; current sophisticated long-term treatments (drugs, pacemakers, stents, etc.) are only palliative. However, in the continued absence of standardized criteria, the injection of cells from different sources and stages of differentiation (skeletal myoblasts, embryonic stem cells (ESC), bone marrow‐derived mononuclear cells (BMMNCs), mesenchymal stem cells, hematopoietic stem cells, endothelial progenitor cells), have not produced consistent results (Menasche, 2018). All experimental and clinical protocols stem from the same basic concepts to isolate and expand the cells that are going to be implanted. The original tissue is dissolved with enzymes capable of damaging the cell membrane, and isolated cells are cultured using procedures (bidimensional, culture media, etc.) consolidated for mature cells. This approach does not consider that optimal implantable cells are secluded in a specific microenvironment (niche) of the native tissue, where their fate is regulated by specific biological and physical signals (Vining and Mooney, 2017). Current protocols neglect stem cell singularity and yield a suboptimal population of somatic stem cells that retain some level of genetic instability. This genetic instability includes tumorigenic and immunogenic properties. Cultured cells also display inadequate purity that may be responsible for graft‐related arrhythmias when transplanted. In most trials aimed at heart repair, MSCs and heart‐derived progenitor cells from cardiospheres or c‐Kit + resident cells (kit + CPCs) are used despite their poor cardiomyogenic potential. Better results are expected when injecting ECS and iPS, but they are not clinically used. Beneficial cell effects could also be ascribed to mRNA transcripts and/or paracrine signals transferred by stem cell-secreted or directly injected exosomes (Gnecchi et al., 2008). This observation has raised the question of whether injecting selected families of exosomes vs. cells could be more efficient in repairing the cardiac tissue’s texture and spur the function of the injured cardiomyocytes. However, recent studies have suggested that the functional improvement in post-MI cardiac function can be attributed to an acute inflammatory-based wound-healing response characterized by the temporal and regional induction of CCR2+ and CX3CR1+ macrophages rather than to the formation of new cardiomyocytes (Vagnozzi et al., 2020) (Figure 2).

### Reconstituting the native bio architecture

Current protocols are focused on cell differentiation in the ischemic region but neglect the need to restore the original myocardial bioarchitecture. The unique spiral-like arrangement of the contractile cells in the myocardium represents the anatomical foundation of the heart’s functional prowess. Instead, after injection into the injured myocardium, stem cells grow and differentiate without a specific polarization; hence, they contribute to the heart contracting in an uncoordinated and inefficient fashion. The issue of the post-injection cell orientation has been addressed by growing cells on polymeric biocompatible structures (scaffolds). These are made of natural, artificial, and composite materials, characterized by a variegated design providing cells with mechanical support to favor a three-dimensional orientation (Reis et al., 2016). Recent data suggests that the decellularization of the tissue can produce innate ECM scaffolds that supply biological signals to the implanted cells. This was demonstrated through successful repopulation with human iPS-derived cardiomyocytes displaying sarcomere structure and electrical conductivity (Taylor et al., 2018). However, this strategy is affected by the inconsistency of different preparations, the possible transfer of viruses and the potential for rejection. Taken together, all scrutinized solutions do not allow the replication of myocardial architecture.

Further knowledge must be developed on the ECM structure and the complex array of biological and physical signals interlacing scaffolds and cells. In this context, an auspicious research direction is represented by the emulation of the ECM structure (microfibers embedded in a matrix). Experimental scaffolds made of a woodpile structure embedded in hydrogel are already under investigation, and have shown promising preliminary results (Carotenuto et al., 2020). However, the exploitation of different scaffold designs has taught us that cell fate can be addressed through specific signals from the nucleus affecting the stiffness of ECM. Translation occurs via the cytoskeleton and via a biochemical cascade modulated by the TAZ/YAP system (Brusatin et al., 2018). Expanding knowledge on signals that lead to ECM stiffness could be fundamental to designing clinical-grade scaffolds.

### Managing the recipient tissue microenvironment

Another factor for successful cell therapy is the modulation of the turmoil microenvironment in damaged tissue. This can hamper its response to humoral/immunostimulant factors or alien cell integration. Deprivation of blood supply modifies tissue pH, while cell debris, tissue edema, and cell misalignment disrupt the signals related to the stiffness of the recipient myocardial tissue. Furthermore, ECM breakdown products, and mitochondrial DNA activate a robust inflammatory reaction, while the invasion of non-myocardial cells creates an ecosystem unfavorable for living cells. In this context, chemokines mobilize monocytes that transdifferentiate into macrophages releasing pro‐inflammatory cytokines (TNF, IL‐1β, and IL‐6) detrimental to surviving cardiomyocytes. These inflammatory factors stimulate fibroblast proliferation, enhancing scar tissue formation to substitute dead cardiomyocytes and prevent ventricular wall rupture. At the same time, the anti‐inflammatory M2 macrophages secrete factors that may recruit and activate exogenous or resident progenitor cells. It is crucial to modulate the post-ischemic microenvironment to favor the implant of new healthy cells. An environment in favor of new healthy cells can be achieved by improving the pharmacological treatments currently in use and injecting cell populations able to interact with immune and non-immune cells. MSCs release soluble factors that impair T‐cell proliferation and differentiation, cytokine secretion, and cytotoxic potential. They suppress the formation of TH1 and TH17 while enhancing the formation of TH2 lymphocytes, which produce anti‐inflammatory cytokines, such as IL‐4 and IL‐10 (van den Akker et al., 2013). In addition, MSCs suppress neutrophils, dendritic cells, and natural killer (NK) cells (Raffaghello et al., 2008; Hamid and Prabhu, 2017) which induces the conversion of T cells into T‐regulatory cells (Di Ianni et al., 2008). These T cells have cardioprotective and regenerative effects that enhance macrophage differentiation into the M2 subtype. This subtype reduces proinflammatory cytokine production, and stimulates cardiac reparative pathways, anti‐inflammatory mediators and angiogenesis (Gore et al., 2015). MSC and CPC-released exosomes can activate post-ischemic modulation of inflammatory and immune responses. Such modifications include the polarization of M1 to M2 macrophages via shuttling miR-182 (Zhao et al., 2019). Thus, exosomes could be used as immunomodulating agents of the myocardial environment to determine post-ischemic conditions more suitable to allow engrafted cells to grow, differentiate and integrate into the recipient surrounding tissue.

### Artificial mitochondrial transfer

Mitochondria transfer is one of the biological processes triggered by stress signals, during which mitochondria are transported from healthy donor cells and incorporated into the endogenous mitochondrial network of the damaged recipient cell, in order to repair damage and restore its bio-energetic profile and health (McCully et al., 2022; Wang et al., 2022). As mitochondrial transfer has been found to play a critical role in healing several pathological conditions, AMT has recently emerged as a promising therapeutic approach for numerous disorders characterized by mitochondrial damage, including ischemic injury, which commonly complicates organ transplantation. A first-human clinical study was performed in pediatric patients in critical conditions due to severe myocardial ischemia–reperfusion injury (Emani et al., 2017; Emani and McCully, 2018; Guariento et al., 2021). The patients received autologous mitochondria isolated from their own rectus abdominis muscle. Mitochondria were administered via multiple injections directly in hypokinetic areas of the myocardium. While no adverse side effects were noted, patients receiving AMT had a more rapid and robust return of systolic ventricular function (McCully et al., 2022).

## Liver

### Cell therapy

Different cell therapies and bio artificial livers have been attempted and used not only for advanced cirrhosis but also for: Acute and acute-on-chronic liver failure, inborn errors of metabolism, chronic cholestatic, autoimmune diseases, and non-alcoholic fatty liver disease (NAFLD, proposed new acronym MAFLD) (Struecker et al., 2014; Qi et al., 2015; Giancotti et al., 2019). Hepatocyte transplantation represents proof of the concept of liver cell therapy. Indeed, clinical observations have demonstrated the procedure’s safety, and patients (∼100) have shown transitory clinical improvement and/or partial correction of the underlying metabolic defect (Lee et al., 2018). The major challenges associated with hepatocyte transplantation include the limited supply of donor organs to isolate good quality cells, low cell engraftment, cryopreservation difficulties, and the necessity of long-term immunosuppression. Advanced grafting strategies have the potential to improve the outcome of hepatocyte transplantation (Puppi et al., 2012).

MSCs derived stem cells, including bone marrow hematopoietic stem cells (HSCs) (CD34 and CD133) and MSCs (CD105, CD73, and CD90), are autologous, easily sourced, and readily cryopreserved, allowing for transplantation procedures with minimal, if any, complications (Forbes and Newsome, 2012; Moore et al., 2014). However, while clinical outcomes occurred within days to weeks, long-term effects (after more than a few months) were not observed. A recent multicenter phase-II open-label controlled trial of HSCs was completed in which repeated autologous infusions of G-CSF-mobilized CD133 + cells were administered to patients with advanced cirrhosis (versus conservative management or treatment with G-CSF alone) (Newsome et al., 2018). Researchers found no impact on liver function or fibrosis. Most recently, it has been shown that leukapheresis and macrophage infusion were well tolerated (Moroni et al., 2019). Tissues are highly informative, especially when clinical results are weak or absent (Lanthier et al., 2017). Studies have shown that the role of mesenchymal-derived cells does not depend on repopulation but on the production of factors and cytokines with multiple effects (An et al., 2017; Starkey Lewis et al., 2020).

Human fetal and adult livers contain two stem cell niches—the ductal plates/canals of Hering that contain hepatic stem cells (HpSCs) (Schmelzer et al., 2007) and the peribiliary glands that contain biliary tree stem/progenitor cells (BTSCs) (Cardinale et al., 2011). In a controlled trial of subjects with decompensated liver cirrhosis receiving fetal EpCAM + HpSC infusion via the hepatic artery, there was a significant decrease in patient MELD scores in the treated group (*N* = 25) at the 6-month follow-up (Khan et al., 2010). In Western countries, Pietrosi et al. treated nine patients by intrasplenic infusion of total fetal liver cell population and demonstrated positive effects on clinical scores and encephalopathy (Pietrosi et al., 2015). Preliminary results have been reported for a phase I/II clinical trial consisting of fetal BTSCs transplantation via the hepatic artery in patients with advanced cirrhosis (Cardinale et al., 2014). Remarkably, in all trials employing fetal liver-derived stem cells, immune suppression was not required even though donors and recipients were not matched for histocompatibility antigens.

Embryonic stem cells evoke ethical concerns. Significant advancements have been made in defining protocols for the differentiation of human-induced pluripotent stem cells (iPSCs) into functional mature hepatocytes, e.g., induced multipotent progenitor cell-derived hepatocytes (Zhu et al., 2014), the direct reprogramming of fibroblasts or MSCs (Huang et al., 2014a; Du et al., 2014; Rezvani et al., 2016), and the utilization of human gastric epithelial cells differentiated into endodermal progenitors (Wang et al., 2016).

### Liver engineering using ECM-based scaffolds

In 2009–2010, the first experiments involving whole-liver decellularization in rodents were conducted by Baptista et al. (2009) and successively completed by Uygun et al. (2010). Important further steps include the decellularized vascular network of the rodent liver by Wake Forest (Baptista et al., 2011) and the human liver decellularization by Mazza et al. (2015). Large-scale production of primary liver bipotential adult progenitor cells have been obtained through suspension cultures (Schneeberger et al., 2020). Although, Takebe et al. (2013) and Takeishi et al. (2020) biofabricated human livers for transplantation using human hepatocytes, biliary epithelial cells, and vascular endothelial cells; current challenges involve recreating “admirable” vasculatures (portal and arterial) and the biliary tree framework. An alternative approach to manufacturing the whole organ is the manufacturing of smaller organoids. These organoids can be generated from a growing number of sources, e.g., bile duct-derived organoids (Huch et al., 2015; Tysoe et al., 2019; Rimland et al., 2021), extrahepatic bile duct-derived organoids (Lugli et al., 2016; Sampaziotis et al., 2017), gallbladder-derived organoids (Lugli et al., 2016; Rimland et al., 2021), and hepatocyte-derived organoids (Hu et al., 2018). “Liver bud organoids'' were obtained by co-culturing iPSC-derived hepatic endoderm cells, endothelial progenitors, and mesenchymal progenitors (Koike et al., 2019).

## Lung

Chronic respiratory diseases are among the leading causes of death after cardiovascular diseases and cancer, accounting for about 5.7% of total deaths in 2017 (Kochanek et al., 2019). The gold standard for treating patients with end-stage lung disease is lung transplantation. However, the availability of donor’s lungs is minimal compared to the high demand from patients on the waitlist for lung transplantation. Accordingly, stem cell and tissue engineering aim to address this challenge by manufacturing alternative functional lung grafts for transplantation therapy.

The human lung comprises a sizeable epithelial surface interfacing with the external gaseous environment and a dense vascular network surrounding the epithelium, separated by a thin basement membrane. Accordingly, a prerequisite for lung engineering is to identify and develop expandable, patient-derived sources of lung epithelial and endothelial cells that are necessary to reconstruct the gas-exchange function. The lung epithelium comprises the proximal airways and distal alveol (Franks et al., 2008). The airways are lined by pseudostratified epithelium, including basal, secretory, and ciliated cells (Hogan et al., 2014). Basal cells are the stem cells of airways and can differentiate into secretory and ciliated cells (Hong et al., 2004; Rock et al., 2009). Importantly, basal cells can be conveniently obtained from patients using minimally invasive procedures (e.g., endobronchial brushing), and can be expanded extensively *in vitro* (Mou et al., 2012). Therefore, they are an ideal cell source for reconstituting the proximal airway in bioengineered lungs. The distal alveoli are primarily covered by terminally differentiated alveolar type 1 (AT1) cells, which cover 95% of the gas-exchange surface (Crapo et al., 1982; Williams, 2003), and surfactant-producing alveolar type 2 (AT2) cells, which are alveolar stem cells with the potential of differentiation into AT1 cells (Rock et al., 2009; Barkauskas et al., 2013; Desai et al., 2014). While isolation of primary AT2 has been reported in mice (Demaio et al., 2009; Bantikassegn et al., 2015; Sinha and Lowell, 2016), rats (Bundschuh et al., 1995; Chen et al., 2004; Gonzalez and Dobbs, 2013), and humans (Elbert et al., 1999; Witherden and Tetley, 2001; Wang et al., 2007; Ballard et al., 2010; Fujino et al., 2011), these cells have a very limited proliferative capability *in vitro* (Dobbs, 1990). To generate an alternative source of AT2 cells, by recapitulating embryonic lung development, advances in stem cell engineering has made it possible for directed differentiation of human induced pluripotent stem cells (hiPSCs) into definitive endoderm (D'Amour et al., 2005; Loh et al., 2014), anterior foregut endoderm (Green et al., 2011), and lung epithelial progenitors characterized by NKX2.1 expression (Longmire et al., 2012; Mou et al., 2012; Hawkins et al., 2017). Further development employing 3D hydrogel culture has enabled the derivation of surfactant-producing AT2 cells from the hiPSC-derived lung progenitors (Huang et al., 2014b; Chen et al., 2017; Jacob et al., 2017). Recent progress is being made to finally induce AT1 specification from hiPSC-derived AT2 cells (de Carvalho et al., 2019).

An engineered lung is incomplete without proper vascularization. In sharp contrast to the discontinuous endothelium in the liver sinusoid or the fenestrated endothelium in the small intestine, the pulmonary microvasculature plays essential roles in gas exchange. It features a continuous, non-fenestrated phenotype (Marcu et al., 2018). However, the understanding of pulmonary-specific microvascular phenotype remains limited at the molecular level. There is a lack of understanding of signaling that regulates the acquisition of such specialized endothelial phenotype during organogenesis and hiPSC differentiation. Accordingly, most lung bioengineering attempts so far have been focusing on using primary endothelial cells isolated from the lung or other tissues and generic endothelial cells from hiPSC differentiation (Ren et al., 2015; Zhou et al., 2018). Future research should focus on defining the molecular signatures of pulmonary-specific endothelium and developing a strategy for deriving such cells from hiPSCs, as accomplished in deriving blood-brain-barrier endothelium (Lippmann et al., 2012; Neal et al., 2019; Praca et al., 2019).

**FIGURE 3:**
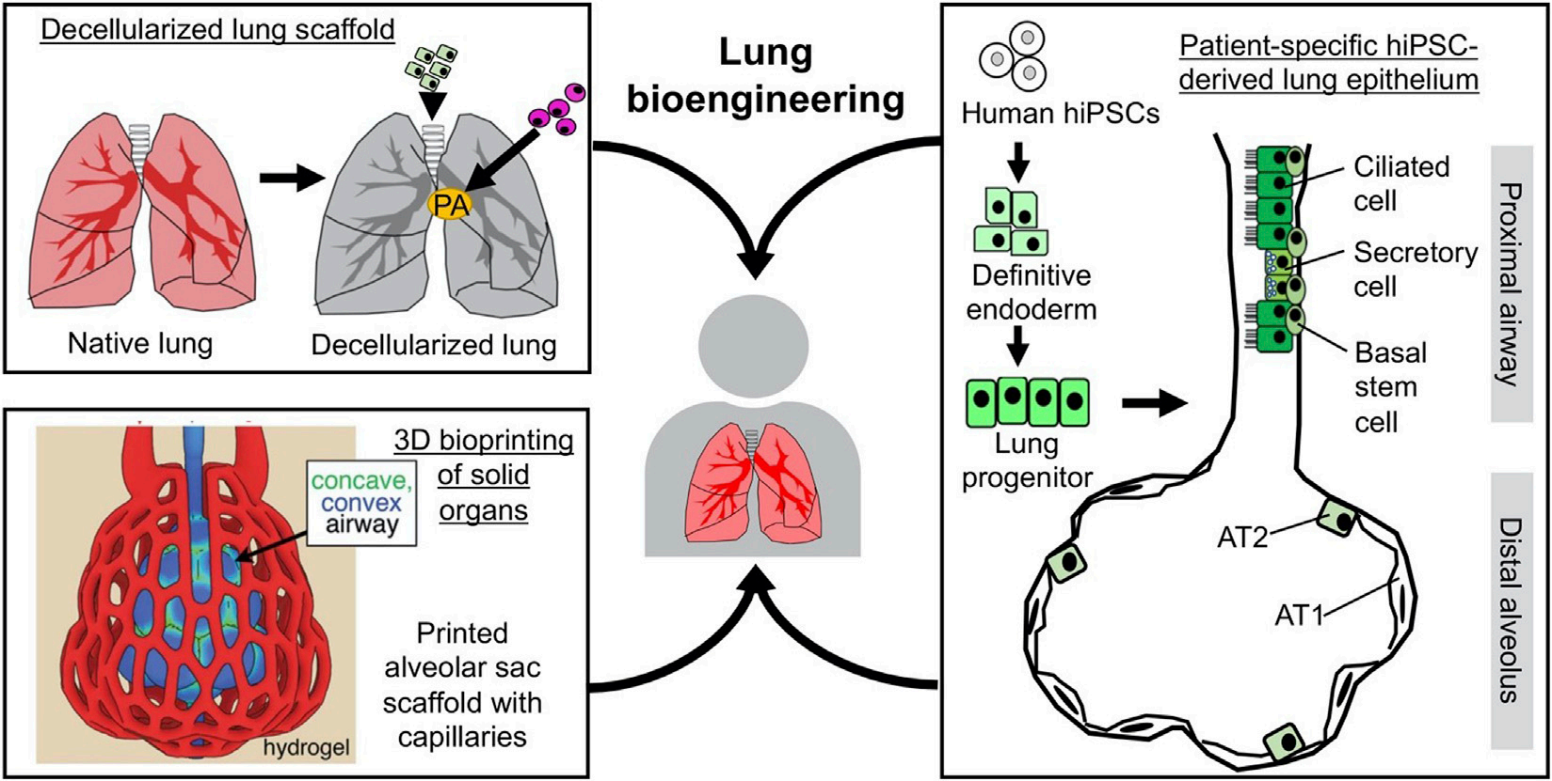
Combining regenerative cells and scaffolds for lung bioengineering. The scaffolds can be derived from whole-lung decellularization and 3D bioprinting (adapted from “Multivascular networks and functional intravascular topologies within biocompatible hydrogels” by Grigoryan et al., 2019, Science, 364 (6439), p 461. Copyright 2020 by The American Association for the Advancement of Science. Reprinted with permission). The cells for reconstituting the lung epithelium can be derived from stepwise differentiation of hiPSCs.

The structural basis of respiratory function lies in the mutually integrated respiratory epithelial and vascular compartments. Biomaterial scaffolds are usually employed to recapitulate such organotypic tissue organization from a tissue engineering perspective. So far, two scaffolding strategies have shown promise in achieving decellularized and 3D-printed lung scaffolds. Whole-lung decellularization uses detergent to remove all the cellular components while preserving the ECM that outlines internal tissue compartmentalization. The decellularized scaffolds enable compartment-specific delivery of pulmonary epithelial and endothelial cells into the airway/alveolar and vascular compartments, respectively (Figure 3). Such strategy has enabled the bioengineering of functional lung tissues that can provide gas-exchange function *in vitro* and *in vivo* (for short term) in small and large animal models (Ott et al., 2010; Petersen et al., 2010; Ren et al., 2015; Nichols et al., 2018; Zhou et al., 2018). In parallel, advances in 3D bioprinting offer an alternative strategy for manufacturing rationally designed tissue scaffolds. In particular, a new bioprinting technique, StereoLithography Apparatus for Tissue Engineering (SLATE) has recently been reported, which used biocompatible food dye additives as potent photo absorbers. It has also enabled 3D printing of hydrogel into biomimetic alveolar models with both gas and vascular compartments (Grigoryan et al., 2019). Comparing the two scaffolding strategies, decellularized scaffolds offer the advantage of preserving the complex, organotypic ECM composition, while bioprinting has so focused on a limited number of ECM molecules, such as collagen and gelatin. On the other hand, in terms of material availability, 3D printing scaffolds could offer, in theory, unlimited supply, while scaffolds manufactured by native organ decellularization still rely on tissue availability (Figure 3).

**FIGURE 4:**
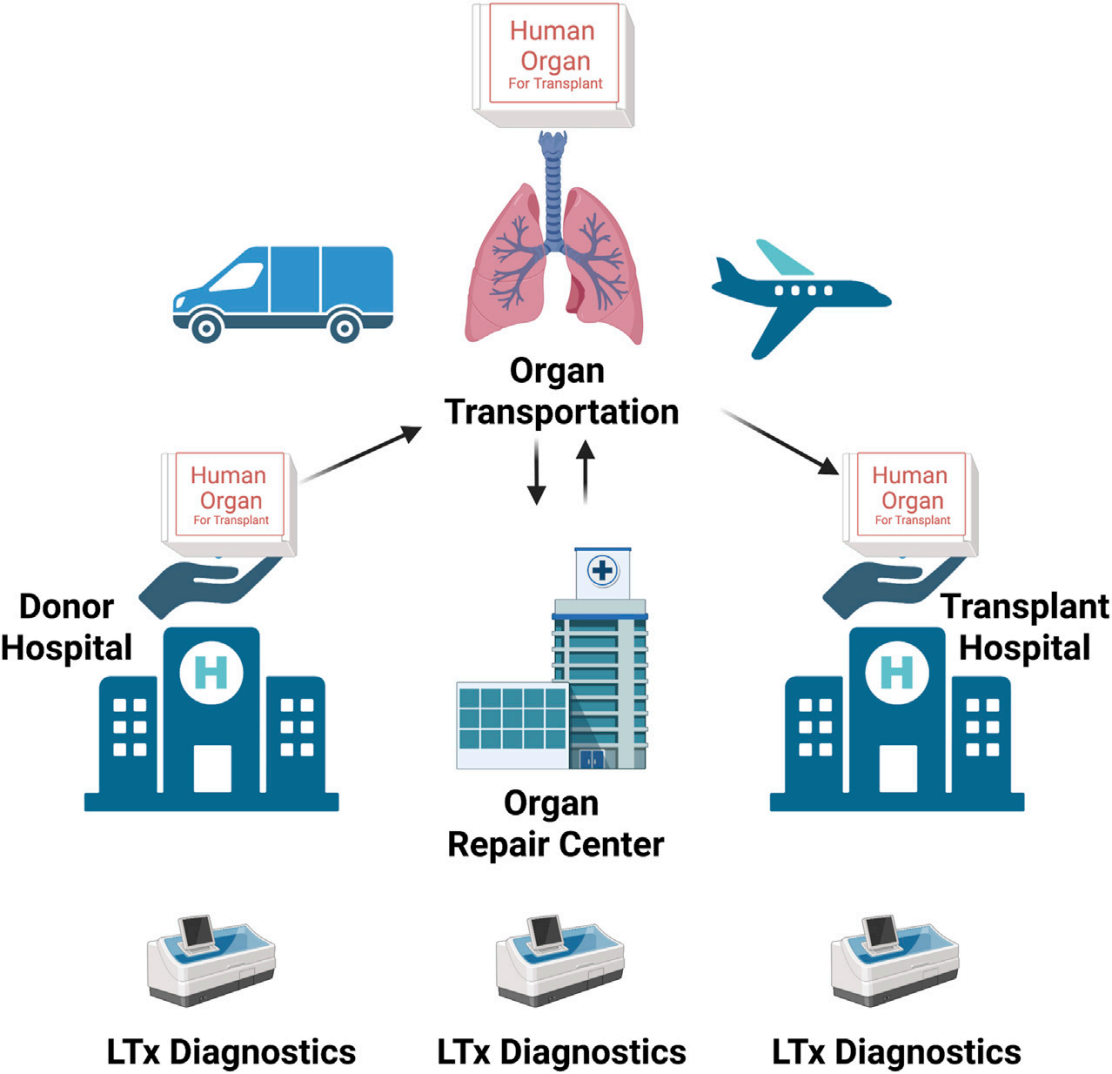
The schematic representation of the mode of operation of the modern “organ-management ecosystem.” Marginal organs are procured at the donor hospital and then transported and delivered to the “Organ Repair Center.” Here, they are subjected to tests to determine whether there is any margin to repair and regenerate them to become transplantable. Once an organ has been repaired and regenerated, it can be transported to the transplant center, where it will be transplanted (adapted from JTCVS Open) (176). Created with BioRender.com.

Upon proper cellularization and *in vitro* maturation, the engineered lung grafts should be evaluated *in vivo*. Considering the inadequate functionality of the lung tissues engineering with current approaches, complete replacement of native lung function in an animal model is usually not feasible. Accordingly, *ex vivo* lung perfusion (EVLP) and heterotopic transplantation models are being developed to bridge to orthotopic lung transplantation. EVLP is a clinically used procedure for normothermic support of donor’s lungs prior to transplantation. Conceived and developed at the Toronto Lung Transplant Program led by pioneering surgeon Shaf Keshavjee (Cypel et al., 2011), the EVLP technology has revolutionized the field of lung transplantation. By repairing and rendering transplantable marginal lung allografts that decades ago would have otherwise been discarded, EVLP has dramatically increased the donor pool and has paved the ground for the development and implementation of a visionary idea, the “Organ Repair Center.” This is a highly specialized transplant unit where organs unsuitable for transplant yet with a decent functional reserve are subjected to diagnostic tests and treatments to make them transplantable. (Figure 4).

In a recent study, the EVLP system was adapted to support partial lung decellularization, re-epithelialization, and functional assessment (Dorrello et al., 2017). To bring EVLP closer to lung transplantation, a xenogeneic cross-circulation model has been developed by connecting the pulmonary vessels of human donor lungs to the circulation of a Yorkshire swine. Such whole-blood cross circulation enabled functional and histological recovery of acutely injured human lungs declined for transplantation (Hozain et al., 2020). These EVLP and cross-circulation models offer promising alternatives to conventional orthotopic transplantation for evaluating and potentially further maturing bioengineered lungs.

## Gastrointestinal tract

The complex cytoarchitecture of the GI tract presents a challenge to generating tissue-engineered GI organs. GI organs are made of a diverse population of cells that collaborate to regulate organ function. For example, while the epithelial layer is responsible for absorptive and secretory functions, it is regulated by the submucosal plexus of the enteric nervous system (ENS) (Bitar and Zakhem, 2013). Choosing the correct combination of cells and scaffolds to recapitulate these functions is difficult. Cell sources may include donor tissue or pluripotent stem cell (PSC)-derived tissue. While donor tissue is obtained from a finite source and can be challenging to expand *in vitro,* multiple cell types in GI tissue can be isolated. PSCs can differentiate into any tissue type and can be generated from donor tissue or obtained from existing cell lines, thus providing a theoretically infinite source of the material. Individual populations of epithelial cells, smooth muscle cells, and ENS cells have all been isolated from donor tissue and generated from PSCs *in vitro*. The cells must be expanded *in vitro* while maintaining their *in vivo* properties, which is complex with individually isolated/PSC-generated cell types. *In vivo*, the cells require interaction with other cell populations to maintain and regulate their phenotype and function (Bitar and Zakhem, 2015). Isolated smooth muscle cells (ISMCs) from rat intestines have shown to develop an altered immature phenotype that favors proliferation over differentiation when cultured *in vitro*. However, when intact strips of smooth muscle are isolated, the smooth muscle cells maintain their mature phenotype and undergo period contraction *in vitro*. Enteric neuronal and glial markers were also present in the smooth muscle strips suggesting that they are required to maintain the correct phenotype and function (Walthers et al., 2014).

Multiple sources of scaffold material have been employed to generate tissue-engineered GI tissue. The mechanical properties of the scaffold must be similar to the native extracellular matrix (ECM) *in vivo* to provide the cells with the appropriate mechanical cues and allow for vascular, lymphatic, and neural ingrowth. Synthetic materials such as polyglycolic/poly lactic-co-glycolic acid (PGA/PLGA) or polyglycolic/poly-L-lactic acid (PGA/PLLA) have readily tunable mechanical properties that have been used to generate scaffolds for GI applications (Basu et al., 2012; Maemura et al., 2012; Rego et al., 2016a; Schlieve et al., 2017). Natural scaffolds such as chitosan (Zakhem et al., 2012; Zakhem et al., 2014; Zakhem et al., 2015; Rego et al., 2016a; Rego et al., 2016b) and acellular ECM (Totonelli et al., 2012; Urbani et al., 2018) provide both mechanical and biochemical cues to the cells and maintains the natural architecture of the tissue. The complex 3D tissue models of intestinal epithelium allow for better mimicking cellular interactions of physiology or pathophysiology and applications towards therapeutic drug screenings and regenerative medicine. Bioengineered small intestine epithelium tissue cultured on lyophilized silk protein sponge matrices with macrophages is a novel system for studying the epithelial-immune interactions reflective of inflammatory bowel disease (Roh et al., 2019). While a more physiological model of the small intestine with a functional epithelial barrier was generated using small intestinal submucosa scaffolds seeded with intestinal organoids obtained from intestinal crypts and co-culture with fibroblasts. After 7 days, a subpopulation of cells differentiated into intestinal-specific cell types such as mucus-producing goblet cells or hormone-secreting enteroendocrine cells (Schweinlin et al., 2016). They have also regenerated intestinal and esophageal tissue (Badylak et al., 2011a; Wong et al., 2011; Kitano et al., 2017).

The lack of transplant options and dismal intestinal transplant survival rates present a significant clinical need for tissue-engineered GI organs; however, GI tissue complexity presents a major challenge to achieving this goal. Recent advances in *in vitro* cell culture, such as the development of organoid systems and the generation of scaffolds that recapitulate *in vivo* organ mechanical and biochemical properties, are promising for the generation of tissue-engineered GI organs.

## The endocrine pancreas

β cell replacement through either whole pancreas or islet transplantation represents the gold standard for the treatment of longstanding morbid diabetes mellitus (Orlando et al., 2014; Odorico et al., 2018). However, its application is limited by a dramatic organ shortage and the need for lifelong anti-rejection medications whose administration is burdened by high costs and morbidity. Since the advent of the regenerative medicine era, it has become apparent that the field of beta cell replacement offers a formidable platform for the application of technologies aiming at either identifying a potentially inexhaustible source of islets or improving islet (immune)protection, lifespan, viability, and function. The present paragraph will focus on this latter task.

Extensive efforts have been focused on developing effective islet encapsulation approaches to eliminate the need for chronic immunosuppression to prevent allograft rejection and recurrence of autoimmunity, for example by designing novel encapsulation materials, and engineering the site of transplantation to improve graft vascularization and provide local immune modulation, in order to overcome some of the aforementioned challenges. Several strategies, including microencapsulation of islets in hydrogel microcapsules (Scharp and Marchetti, 2014; Cantarelli et al., 2017) as well as retrievable macroencapsulation devices (MEDs) (Weaver et al., 2018), have been developed with the objective of providing an immune protective environment to the islets, with each having its own benefits and limitations (Scharp and Marchetti, 2014). Islet encapsulation in hydrogel microcapsules, including alginate capsules, has been shown to provide immunoprotection to the islets overcoming allogeneic, xenogeneic and autoimmune responses. The spherical shape of the microcapsules also maximizes the surface area to volume ratio, resulting in increased diffusion of oxygen and nutrients. However, the major disadvantage of microencapsulation is that the islet capsules cannot be regarded as a single construct but as a multitude of independent microtissues, and it can be challenging to control their localization as well as practical surgical implantation and retrieval (if needed), thereby raising concerns to the biosafety of this approach (Storrs et al., 2001; Weaver et al., 2018). The MEDs can physically isolate the islets from the surrounding environment by a semipermeable cell containment barrier and provide immunoisolation by preventing direct contact with the host (Scharp and Marchetti, 2014). The larger dimensions of the device also allow for easier retrieval in case of adverse events, overcoming a potential regulatory hurdle associated with cell therapy. However, islets entrapped in the device can agglomerate over time, resulting in a larger tissue with nutrient and oxygen diffusion limitations, leading to necrosis and loss of function. If they are embedded in a bulk hydrogel (example, alginate sheet), it can result in a reduced surface-to-volume ratio, leading to difficulties in scaling up to a clinically relevant size, without compromising nutrient and oxygen diffusion. Even with thin planar devices providing a larger surface-to-volume ratio, the upscaling to a therapeutic islet dose remains challenging. Finally, the lack of a pancreas-specific biochemical microenvironment or peri-islet niche can adversely affect the long-term viability and function of islets in both microencapsulation and MED platforms. Therefore, research has focused on developing an islet-specific niche by using mammal organs as a source of 3D ECM scaffolds that is inherently biocompatible and provides biochemical cues and 3D support similar to that of the native tissue environment (Mirmalek-Sani et al., 2013; Peloso et al., 2016; Asthana et al., 2021).

**FIGURE 5:**
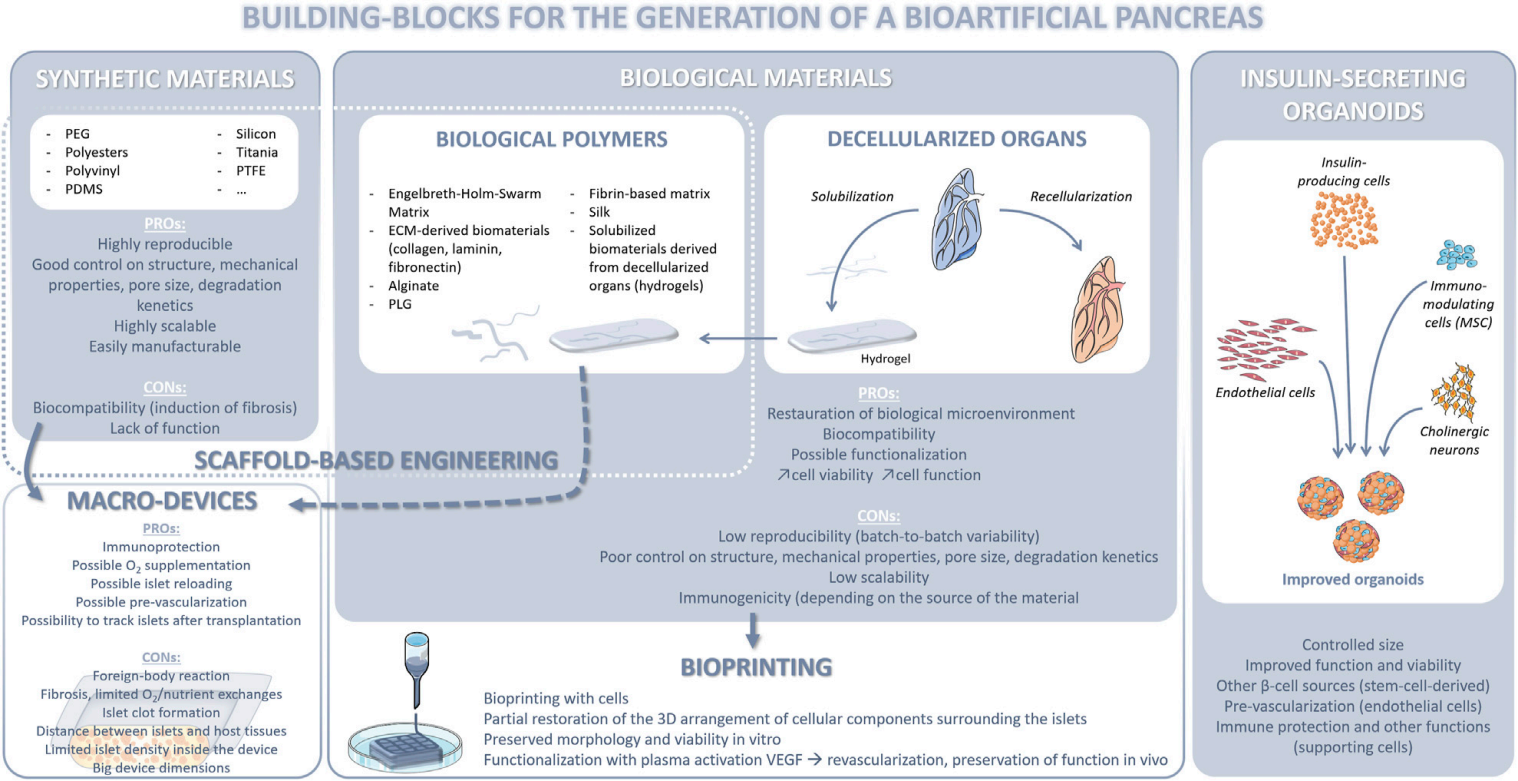
Regenerative medicine strategies applied to beta cell replacement.

Islets have a high oxygen demand, considering the fact that they account for 1%–2% of the pancreatic volume but receive 5%–10% of pancreatic blood flow (Jansson et al., 2016). Therefore, therapeutic islet constructs would require revascularization and integration with the host in order to maintain long-term graft viability and function. In fact, insufficient nutrients and oxygen supply due to the lack of proper revascularization post‐transplantation is a major factor for the loss of transplanted islets and insufficient glucose/insulin diffusion delays glucose sensing and insulin secretion (Bruni et al., 2014). The best way to develop vascularized tissues is still through self-vascularization within bioengineered tissue constructs. However, revascularization is a slow process and the time required for the assembly and maturation of a perfusable vascular network throughout the graft may be longer than its survival time, as tissue necrosis often occurs early during the engraftment period due to insufficient oxygen supply. Therefore, incorporation of biochemical factors that favor rapid vascularization could help reduce islet death and loss of graft function after transplantation. Furthermore, a construct architecture and spatial patterning of islets that reduce their distance from the surrounding body fluid/host vasculature would allow for more efficient diffusion. Maximization of graft surface area would also lead to enhanced oxygen diffusion and promote attachment, proliferation and migration of host vascular cells, which would result in rapid vascularization, tissue remodeling, and prolonged islet survival following transplantation. Incorporation of microchannels in the construct containing islets would facilitate sufficient nutrient/oxygen supply with culture media *in vitro* and stimulate accelerated inosculation with the host vasculature *in vivo*. Such an architecture will ensure efficient nutrient and oxygen diffusion (Cabodi et al., 2005; Stachowiak et al., 2005; Ling et al., 2007) by providing an increased surface-to-volume ratio and extending the diffusion limit, compared to a bulk hydrogel (without microchannels), and promote engraftment, thus preserving islet viability and function in larger therapeutic constructs. Traditional biofabrication techniques including, particulate leaching, solvent casting and electrospinning can generate porous scaffolds; however, these techniques have limited compatibility with hydrogels and limited control over construct architecture, including pore/channel size, geometry, and distribution. Moreover, they require application of temperatures, solvents or other conditions that can adversely affect live cells and often rely on post-fabrication cell seeding, which can result in non-uniform cell distribution and poor cell attachment. Additionally, most bioengineered constructs are manually fabricated and assembled, thus lacking a high degree of reproducibility necessary for commercial scale-up, clinical application and regulation. Additive manufacturing technologies that allow for the precise and reproducible fabrication of large 3D constructs with controlled architecture and islet distribution, are therefore being explored to further improve construct prototypes required for making islet-based therapies a reality (Gurlin et al., 2020; Soetedjo et al., 2021). Overall, success in this field will generate only from a combinatorial approach (Figure 5). This will accelerate the translation of current breakthroughs in scientific research to patients and allow islet transplantation to become a widely applicable treatment for morbid, longstanding diabetes mellitus.

## Vascularized composite tissue engineering

Since the first successful hand transplant (Dubernard et al., 1999), vascularized composite allotransplantation (VCA) has progressed and been applied to the face (Dubernard et al., 2007), penis (van der Merwe et al., 2017), abdominal wall (Giele et al., 2016), and uterus (Brannstrom et al., 2015). Despite the extraordinary technical advances, the need for immunosuppression remains critical to managing chronic rejection, and difficulties still remain for re-transplantation. Moreover, specifically for body parts such as the hand and face, very narrow morphological criteria, in addition to the classical immunological screening, are responsible for a very limited donor/recipient match. Recent advances in the Vascularized Composite Engineering (VCE) approach in the direct line of solid organs’ perfusion-decellularization/recellularization (PDR) is promising towards addressing some underlining concerns. Decellularization of simple and non-vascularized tissues, such as skin and bone, has been extensively described; however, the production of complex and large composite tissue matrices is at its early stage but holds great promise (Badylak et al., 2011b; Orlando et al., 2013; Orlando et al., 2019). The current challenge is to find a correct and versatile decellularizing protocol applicable to each tissue type that presents different sensitivity to decellularizing agents. Additional early VCE studies employing PDR techniques have led to encouraging results in the rat limb and face (Jank et al., 2015; Duisit et al., 2018), porcine ear (Duisit et al., 2017a), human face and ear (Duisit et al., 2017b; Duisit et al., 2017c), human hand (Gerli et al., 2018), or sheep uterus (Tiemann et al., 2020). Produced matrices of various origins, sizes, and complexities show preservation of the ECM and their associated vascular tree to allow partial *in vitro* recellularization and *in vivo* transplantation.

For organ engineering, the next crucial step will be the recellularization and transplantation of the scaffolds to generate a functional and sustainable graft. In addition to the number of cells needed to repopulate the ECM, VCE has to address several issues due to tissue types and functions to be restored, i.e., motility, sensation, and the need for very specific bioreactors to be developed, allowing skin/mucosa regeneration and muscular training. The advantage of VCE, compared to SOE, is that a complete *in vitro* recellularization is not obligatory. The organ does not need to be fully functional at the time of implantation, and nearby cell repopulation at the implantation site may also occur, thus highlighting that the recipient can serve as their optimized bioreactor, as advocated by Badylak (Badylak, 2016). Therefore, an adapted approach should find an adequate balance between *in vitro* bioreactor culture and complementary *in vivo* maturation and healing.

## Organoids

**FIGURE 6:**
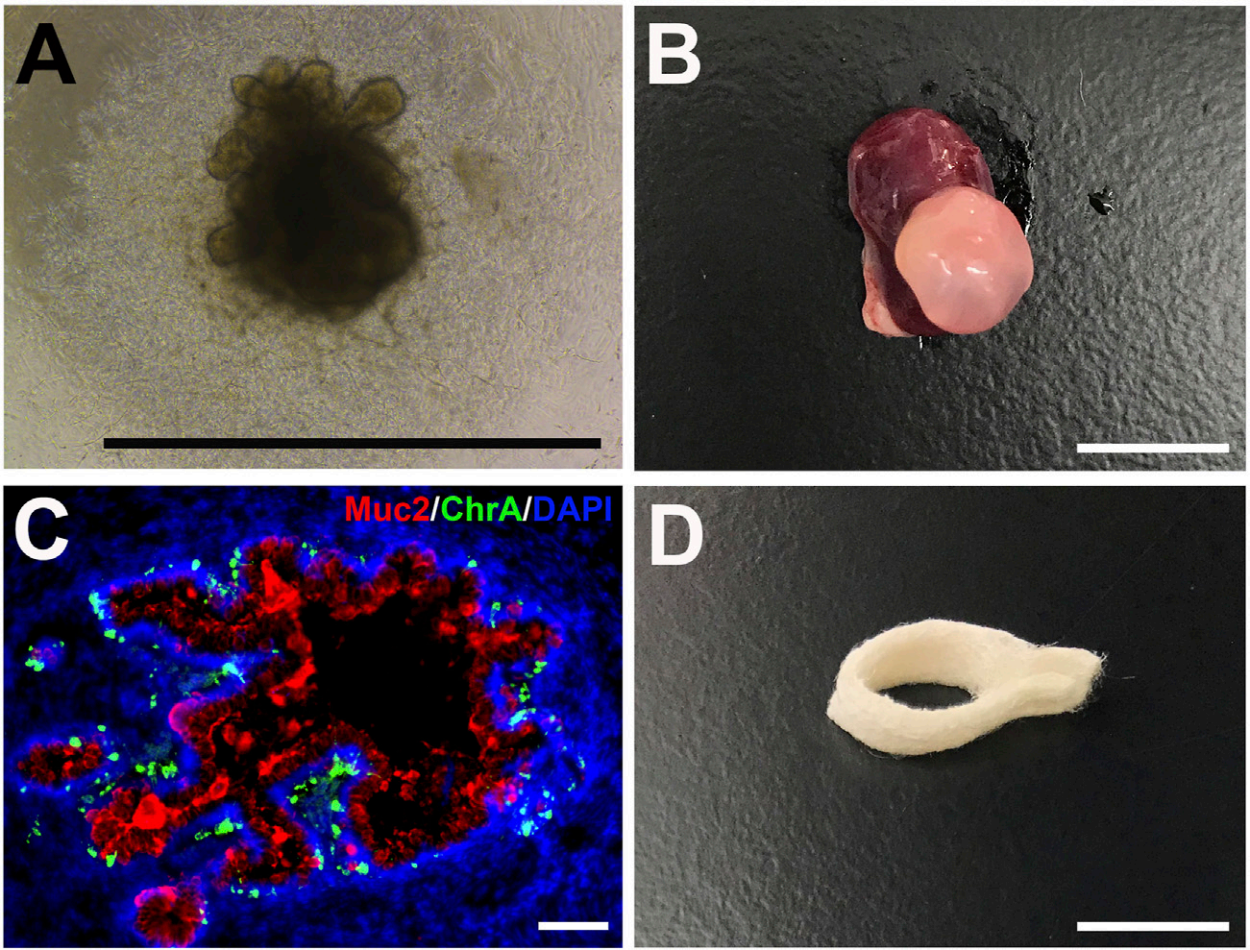
Human intestinal organoids (HIOs) and biodegradable scaffolds. **(A)** Brightfield photomicrograph of a PSC-derived HIO after 25 days *in vitro* (scale bar 1 mm) **(B)** Gross photo of a transplanted HIO (tHIO) after 8 weeks *in vivo* (scale bar 1 cm) **(C)** Immunofluorescent staining of an 8 week-old tHIO for goblet cells (Muc2/Cy5) and enteroendocrine cells (ChrA/FITC) (scale bar 100um) **(D)** Polyglycolic acid and poly-L-lactic acid (PGA/PLLA) biodegradable scaffold can be seeded with cells for the generation of tissue-engineered GI organs (scale bar 1 cm).

Organoids are miniature self-organized 3D structures composed of one or more cell types that partially recapitulate the structure and function of tissues and organs. Organoid technology has emerged as a powerful tool for studying organ development, disease progression, and drug screening (He et al., 2020). Organoid cultures represent an essential advancement in tissue engineering and can better recapitulate *in vivo* conditions *in vitro*. Various cell types such as human-induced PSCs, embryonic stem cells (hESCs), cell lines, and primary cells with or without bioreactor or biomaterials/scaffolds such as PLGA or Matrigel are used to generate complex 3D organoids structures that mimic their *in vivo* counterparts. Large and small intestinal, esophagus and stomach organoids have been generated from donor tissue or PSCs (Figures 6A,B) (Spence et al., 2011; Kasagi et al., 2018; Broda et al., 2019; Lau et al., 2019). Organoids are multicellular spheroids with diverse cell populations that mimic their respective organs’ organization (Figure 6C). This ability allows the cells to maintain the correct phenotype and function *in vitro*. Organoids can be designed in an organ region-specific manner, such as that of the different brain compartments (Qian et al., 2016). Multipart protocols are also designed to incorporate various growth factors, cytokines, and small molecules to promote sequential differentiation of PSCs. Knowledge of developmental mechanisms and PSCs differentiation has allowed the development of multilineage organoids such as the kidney. Takasato et al. (2015) show the generation of kidney organoids through the differentiation of PSCs in 2D and 3D cultures using various concentrations of molecules at different durations and time. Organoid cultures are a powerful tool for studying organ development and function in healthy and disease states and potentially generating transplantable tissue. However, since large amounts of organoid tissue are necessary for a transplant, suitable scaffold (Figure 6D) and culture techniques are needed for further advancement (Finkbeiner et al., 2015). Additional limitations of *in vitro* organoids are the inability to generate mature and diverse cellular structures, their inconsistent reproducibility, and the deficiency of surrounding vascular, nervous, and immune systems necessary to recapitulate the *in vivo* tissue interaction. Limitations also exist in generating vascularized and architecturally organized organoids to mimic their *in vivo* organ counterparts precisely and efficiently. 3D bioprinting has been proposed to address and resolve some of these issues and accelerate the generation of complex organoids (Keshavije, 2020; Ren et al., 2021). Thus, in the future, the incorporation of knowledge in stem cell and developmental biology and advancements in material technology such as 3D printing will promote the development of improved organoids for tissue engineering and disease modeling that better mimic their *in vivo* counterparts both structurally and functionally.

## Final remarks

In the past few decades, regenerative medicine has provided evidence that technologies like decellularization, 3D bioprinting, cell and organ engineering, and blastocyst complementation may offer platforms for the bioengineering, repair, and regeneration of transplantable organs. Although emerging data is promising, the complexity of solid organs poses a significant challenge, and further research and substantial investments are needed, as well as a synergic collaboration among all stakeholders, namely scientists, academia, industry, funding agencies, and governmental institutions. Advancements in basic research knowledge of stem cells, biomaterial, and developmental biology in combination with various biotechnologies and bioreactors are crucial for fast growth. Development and incorporation of chip technologies, organoids, and 3D bioprinters are opening new avenues and directions for the future. As it is clear to us that “no field in health sciences has more interest than organ transplantation in fostering progress in regenerative medicine because the future of no other field more than the future of organ transplantation will be forged by progress occurring in regenerative medicine” (Orlando et al., 2019), we therefore infer that transplant medicine should accept the challenge and lead the efforts that will eventually deliver organs manufactured from patient’s own cells to the bedside.
